# Antihistaminergic and Anticholinergic Properties of the Root Bark Aqueous Extract of *Diospyros mespiliformis* (Ebenaceae) on Hypersecretion of Gastric Acid Induced in Wistar Rats

**DOI:** 10.1155/2022/5190499

**Published:** 2022-01-31

**Authors:** Vandza Luc Vandi, André Perfusion Amang, Christophe Mezui, Gaël Tchokomeni Siwe, Gustave Lebeau Otto Ndji, Hacheked Mbida, Odile Baponwa, Paul Vernyuy Tan

**Affiliations:** ^1^Department of Biological Sciences, Faculty of Science, University of Maroua, P.O. Box 814, Maroua, Cameroon; ^2^Department of Biological Sciences, Higher Teachers' Training College, University of Yaoundé I, P.O. Box 047, Yaoundé, Cameroon; ^3^Department of Animal Biology and Physiology, Faculty of Science, University of Yaoundé I, P.O. Box 812, Yaoundé, Cameroon; ^4^Department of Life Science, Higher Teachers' Training College, University of Ngaoundéré, P.O. Box 652, Bertoua, Cameroon

## Abstract

**Objective:**

The objective of this study was to elucidate the antisecretory mechanism of the root bark aqueous extract of *Diospyros mespiliformis* (RBAEDM) in Wistar rats. *Materials and methods*. RBAEDM was tested on three experimental animal models of gastric acid hypersecretion including pyloric ligation (PL), PL with histamine, and carbachol pretreatments. The ulcerated surface, mucus mass, pH, gastric acidity, and pepsin activity were determined. Some bioactive compounds revealed by qualitative phytochemistry were quantified. Some markers of oxidative stress *in vivo* such as malondialdehyde (MDA), superoxide dismutase (SOD), catalase (CAT), reduced glutathione (GSH), and *in vitro* antioxidant tests (ABTS: 2,2′-azinobis-3-ethylbenzothiazoline-6-sulfonic acid, DPPH: 2,2-diphenyl-2-picrylhydrazyl, and FRAP: ferric reducing antioxidant power) were determined.

**Results:**

In the three models studied, RBAEDM resulted in increases in the percentages of inhibition ranging from 9.50 to 59.52% of gastric ulcer and mucus mass. This increase was accompanied by the reduction in acidity and pepsin activity. The administration of RBAEDM resulted in a significant decrease (*p* < 0.05, *p* < 0.01) in MDA levels correlated with a significant increase (*p* < 0.05, *p* < 0.01) in CAT and nitrite levels compared with the negative control. RBAEDM has the ability to scavenge ABTS and DPPH radicals and to reduce FRAP, and the inhibitory concentration of 50% (IC_50_) of the ABTS radical was 220 *μ*g/mL compared with the butylhydroxytoluene (BHT) control (175 *μ*g/mL). Quantitative phytochemistry revealed abundant polyphenols, flavonoids, tannins, saponins, and anthocyanin.

**Conclusion:**

RBAEDM protected gastric mucous membrane for gastric acid by mechanisms that would involve both anticholinergic and antihistaminergic pathways.

## 1. Introduction

Stress, whether psychological, physical, or physiological, is a highly plausible factor in the development of ulcer disease and is associated with increased morbidity and mortality [1, 2]. Psychological distress is generally correlated with the genesis of gastric ulcers and affects all individuals regardless of their status [2, 3]. Stress induces ulcers through several pathophysiological mechanisms including gastric acid secretion by stimulation of the vagus nerve and ischaemia resulting from hypersecretion of catecholamines [4, 5]. Indeed, vagus nerve stimulation leads to gastric acid secretion, which activates pepsin and produces free radicals such as the superoxide anion, hydrogen peroxide, and hydroxyl radicals. The increased production of the latter causes lipid peroxidation and consequently gastric lesions [3, 5, 6]. Hypersecretion of catecholamines resulting from the activation of the sympathetic-adrenal-medullary axis contributes to ischaemia of the gastric mucosa following vasoconstriction [1]. However, the body possesses its own means of defense such as mucus and bicarbonate secretion, increased activity of antioxidant enzymes, and nitrogen monoxide (NO) synthesis.

The first line of gastric mucosal defense consists of the mucus and bicarbonate barrier. The secretion of bicarbonate into the mucus gel layer is essential to maintain a pH gradient at the epithelial surface, which represents a line of defense against gastric acid [7, 8].

The vasodilating effect of NO contributes in maintaining the integrity of the gastric mucosal barrier [9] through the action of the constituent NO synthase (cNOS). It degrades into nitrite (NO_2_-) and then nitrate (NO_3_). NO is a molecule with a half-life of a few seconds released by vascular endothelial cells. It is synthesized in response to various substances such as histamine and acetylcholine [10]. In the gastrointestinal tract, the NO produced diffuses rapidly into the tissues, where it activates guanylate cyclase, which converts GTP into cyclic GMP. This leads to a decrease in Ca^2+^ concentration and induces relaxation of the smooth muscle and an increase in blood flow [10].

The conventional treatment used for the prevention and healing of stress-induced ulcers is the intake of antisecretory agents such as anticholinergics (verapamil), H_2_ receptor antagonists (ranitidine), proton-pump inhibitors (omeprazole), and antacids (sodium bicarbonate). However, these antisecretory agents have many side effects, including diarrhoea, nausea, constipation, and headache [11]. Hence, the use of phytotherapy is the alternative route for the treatment of gastric ulcers.

*Diospyros mespiliformis* is a plant of the Ebenaceae family used in ethnomedicine for the treatment of ulcers, diarrhoea, malaria, and fever [12]. In addition, its numerous pharmacological properties such as its analgesic and anti-inflammatory [13], antibacterial [14], antiseptic [15], and gastroprotective [16] activities have been demonstrated. However, its antisecretory activity had not yet been studied; hence, the objective of this work was to determine the antisecretory activity and mechanism of action of RBAEDM against gastric ulcers induced by pyloric ligation in rats.

## 2. Materials and Methods

### 2.1. Animal Material

Male albino Wistar rats, 12 ± 2 weeks old and weighing 160 to 200 grams, were used. These animals were bred in the animal house of the Laboratory of Animal Physiology and Pharmacognosy of the University of Maroua. They were fed a standard laboratory diet with the following composition: maize meal (50%), soybean meal (20%), fish meal (15%), bone meal (4%), vitamin complex (0.1%), cottonseed cake (10%), palm oil (0.1%), and cooking salt (0.8%) with unrestricted access to tap water.

### 2.2. Plant Material

The mature adult plants were collected in the forest. The mature root bark of *Diospyros mespiliformis* depth at 10 cm was harvested in the Mokolo locality, Far North of Cameroon (N10°44′12.93072″; E13°47′3.74784″; latitude: 10.73693). The plant was authenticated at the Herbarium Faun School of Garoua in comparison with the existing specimen (No. HEFG/01404). After harvesting, the root barks of *D. mespiliformis* were shade-dried and powdered for extract preparation.

### 2.3. Preparation of RBAEDM

Three hundred grams (300 g) of powder was macerated in 3 liters of distilled water for 24 hours. After filtration using Whatman Paper No. 3, the solution was evaporated in an oven at 50°C, resulting in 12 g of extract (4% yield). The resulting extract was stored at 4°C for further use.

### 2.4. Reagents

Pepsin, histamine, carbachol, NaNO_2_, BSA, NAOH, naphthylenediamine (NED), sulfamide, ABTS, DPPH, and FRAP are purchased from Sigma Chemical and Sigma-Aldrich Chemie (USA, Germany, India).

### 2.5. Quantitative Phytochemical Analysis of RBAEDM

#### 2.5.1. Dosage of Total Phenolic Compounds

The protocol of [17] was used to determine the level of phenolic compounds in the extract. The absorbance was read at 750 nm with a spectrophotometer. The concentration of phenolic compounds was calculated from the regression equation of the gallic acid calibration curve (0–250 *μ*g/mL) and expressed in milligram equivalents of gallic acid per hundred grams of crude extract (mEqGA/100g dry matter).

#### 2.5.2. Dosage of Flavonoids

The total flavonoid content of the extract was determined using the aluminum chloride colorimetric method [18]. The absorbance was measured with a spectrophotometer at 415 nm. The total flavonoid content was calculated using the quercetin calibration curve (0–250 *μ*g/mL), and the results were expressed as milligram quercetin equivalents per hundred grams of extract (mEqQ/100g dry matter).

#### 2.5.3. Dosage of Tannin

The tannin content was measured by the Folin-Ciocalteu method described by [19]. The absorbance was measured with a spectrophotometer at 700 nm. A calibration curve was plotted using tannic acid (0–250 *μ*g/mL), and the results were expressed in milligram equivalents of tannic acid per hundred grams of extract (mEqTA/100g dry matter).

#### 2.5.4. Dosage of Saponins

The quantification of saponins was carried out using the method described by [20]. The absorbance was measured with a spectrophotometer at 530 nm. A calibration curve was drawn using galactose (0–250 *μ*g/mL), and the results were expressed in milligram galactose equivalents per hundred grams of extract (mEqG/100g dry matter).

#### 2.5.5. Dosage of Anthocyanins

The total anthocyanin content was determined using the pH differential method described by [21]. The absorbance was measured with a spectrophotometer at 520 and 700 nm at pH 1.0 and 4.5, respectively.

### 2.6. Antisecretory Screening of RBAEDM

For antisecretory screening, the method described by [22] was used to induce gastric ulcers. After a 48-hour non-hydric fasting, 30 rats were divided into 6 groups (1 normal control, 1 negative control, 1 positive control, and 3 test groups) of 5 rats each. These animals received distilled water (5 mL/kg) for the normal and negative controls, ranitidine (50 mg/kg) for the positive control, and extract (100, 200, and 400 mg/kg) for the test groups. The doses of extract were chosen based on the previous work [16]. One hour after administration of respective treatments, all the animals except normal control underwent a laparotomy performed under anesthesia using ketamine (50 mg/mL) at dose of 2 mL/kg of body weight by intraperitoneal route and associated diazepam (5 mg/mL) at dose of 5 mg/kg. The stomach was ligated at the level of the pyloric sphincter and was carefully replaced into the abdomen, which was then sutured. The animals were deprived of water during the postoperative period. Six (6) hours after ligation, they were again subjected to diazepam/ketamine anesthesia and sacrificed. Stomach was removed from the abdominal cavity, and the gastric content of each rat was collected in dry tubes. The content was centrifuged at 2000 rpm for 10 minutes, the supernatant was collected, and its volume was measured. The gastric juice obtained was used immediately for pH determination and acid titration. Ulcerations were measured lenghtwise and widthwise, and their surface is calculated accordingly for score attribution as described below. The mucus produced in each stomach was carefully scraped off with a microscope slide and weighed on a precision electronic balance. Stomach homogenates (20%) were prepared using phosphate-buffered saline (PBS) for the determination of oxidative stress parameters *in vivo*. The two most active doses during the pyloric ligation test were chosen for antihistaminergic and anticholinergic studies.

### 2.7. Antihistaminergic Activity of RBAEDM

For antihistaminergic activity, the protocol described by [4] was used. Twenty rats were divided into 4 groups of 5 rats each. The positive and negative controls were given ranitidine (50 mg/kg) and distilled water (5 mL/kg) *per os*, respectively. The test groups received extract at 200 and 400 mg/kg. Thirty (30) minutes after their respective treatments, pyloric ligation was performed as described by [22]. Histamine (2.5 mg/kg) was administered subcutaneously one hour after pyloric ligation. Four hours after histamine injection, all the animals were sacrificed, the remaining protocol being the same as described for antisecretory screening.

### 2.8. Anticholinergic Activity of RBAEDM

The protocol was the same as described for antihistaminergic activity with the difference that the positive control group received verapamil (50 mg/kg) instead of ranitidine and histamine that were replaced by carbachol (1 mg/kg) in all groups.

### 2.9. Ulcerated Surface and Ulcer Index

Ulcerated surface and ulcer index were calculated as described by Tan et al. [23]. Ulcerated surface: length *x* width. Ulcer scores were allotted as follows: no ulcer = 0.0; ulcer surface ≤0.5 mm^2^ = 1; ulcer surface >0.5 ≤ 2.5 mm^2^ = 2; ulcer surface >2.5 ≤ 5 mm^2^ = 3; ulcer surface >5 ≤ 10 mm^2^ = 4; ulcer surface >10 ≤ 15 mm^2^ = 5; ulcer surface >15 ≤ 20 mm^2^ = 6; ulcer surface >20 ≤ 25 mm^2^ = 7; ulcer surface >25 ≤ 30 mm^2^ = 8; ulcer surface >30 ≤ 35 mm^2^ = 9; and ulcer surface >35 mm^2^ = 10. The ulcer index (UI) was calculated with the following formula:

(1) $$\begin{array}{c} \text{UI}=\frac{1}{n}\sum_{1}^{n}\text{score}\pm \text{SEM}. \end{array}$$

### 2.10. pH Measurement and Acidity Titration of RBAEDM

The pH was determined using a pH meter. The gastric acidity was measured using the colorimetric titration method by adding two drops of phenolphthalein with the NaOH solution (0.1 N) until the pink coloration was obtained. The volume of NaOH used was recorded to determine the acidity.

### 2.11. Determination of Pepsin Activity of RBAEDM

The determination of the hydrolytic activity of pepsin was performed by incubating 1 mL of gastric juice in 2 mL bovine serum albumin (BSA) (50 mg/mL) at 37°C for 10 min [24].

### 2.12. *In Vivo* Antioxidant Activity of RBEADM on Pyloric Ligature

*In vivo* antioxidant capacity of the extract was evaluated in the stomach homogenates. The determination of total protein was done according to the Biuret method [24] and that of the MDA level was done according to the protocol of Wilbur et al. [25]. The activity of superoxide dismutase (SOD), CAT, and reduced glutathione (GSH) was determined according to the protocol of Misra and Fridovich, Sinha, and Ellman [26–28], respectively.

### 2.13. Determination of Nitrite

Nitrite in stomach homogenates was measured with the Griess reagent according to the method described by [29]. The chromophore absorption during nitrite deionization with sulfanilamide coupled to naphthylenediamine (NED) was read at 546 nm. The product obtained was proportional to the amount of nitrite present in the sample. The nitric oxide level was determined from the calibration curve established from different concentrations of NaNO_2_.

### 2.14. Antiradical Activity of the ABTS Extract

The free radical activity of ABTS (2,2′-azinobis-3-ethylbenzothiazoline-6-sulfonic acid) was measured according to the method described by [30]. Fifty microliters (50 mL) of extract or standard was added to 150 *μ*L of ABTS^+^ (7 mM), and the mixture was stirred and incubated at room temperature for 40 min. The absorbance was read with a spectrophotometer at 745 nm. The antioxidant capacity of the sample was determined from the calibration curve established with Trolox (0–125 *μ*g/mL). The inhibition percentage was calculated using the following formula: *I*% = ((Ac – At)/Ac)  −  100 with (Ac: absorbance of the control, At: absorbance of the test). From this percentage, the concentration of extract inhibiting 50% (IC_50_) of the ABTS radical was determined.

### 2.15. Evaluation of the Antiradical Activity of the Extract with DPPH

This method is based on the measurement of antioxidant ability to trap the DPPH radical [31]. The antioxidant capacity of samples was determined from the calibration range established with the Trolox (0–125 *μ*g/mL).

### 2.16. FRAP Assay

The reducing capacity of the extract was determined according to the method described by Benzie and Strain [32]. The absorbance of the reaction medium was determined at 593 nm. An increase in absorbance corresponded to an increase in the reducing capacity of the tested extract. The reducing capacity of the sample was determined from a calibration range established with vitamin C (0–125 *μ*g/mL).

### 2.17. Statistical Analysis

The data were analyzed using GraphPad Prism 5.03 software. Statistical analysis was performed by the one-way analysis of variance (ANOVA) followed by the Newman–Keuls posttest. The values were expressed as mean ± standard error to the mean (SEM). *p* values <0.05 were considered statistically significant.

## 3. Results

### 3.1. Quantitative Phytochemical Analysis of RBAEDM

The results in Table 1 reveal the presence in RBAEDM of a quantity of certain bioactive compounds (polyphenols (86.58); flavonoids (55.22); tannins (21.71); anthocyanins (10.14); and saponins (21.92) (mEq/100g of dry)).

### 3.2. Effect of RBAEDM on Acid Secretion and Gastric Ulcers Induced by Pyloric Ligation

**Figure 1:**
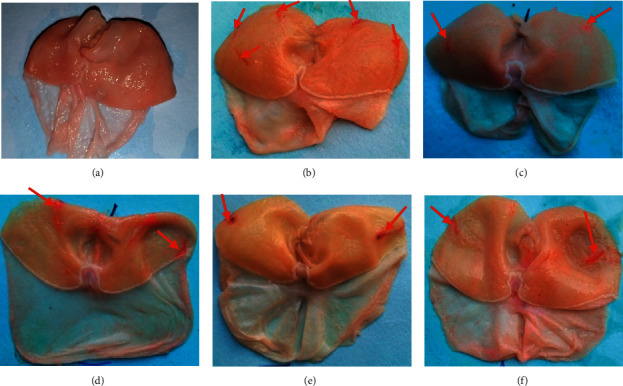
Photograph of stomach ulcerated by pyloric ligation. (a) Normal control; (b) negative control; (c) 100 mg/kg of RBAEDM; (d) 200 mg/kg of RBAEDM; (e) 400 mg/kg of RBAEDM; and (f) 50 mg/kg of ranitidine; 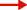: indications of gastric lesions.

Macroscopic observation of the stomach following pyloric ligation in rats shows lesions in the form of red bands on the glandular part of the stomach (Figure 1). Photograph A shows normal healthy mucosa (normal control). The ulcerated surface is larger in the negative control (Photograph B: 22.40 mm^2^) and decreases significantly in animals treated with the extract at doses of 100, 200, and 400 mg/kg (Photograph C: 5.60; Photograph D: 8.80; and Photograph E: 7.60 mm^2^, respectively). Pyloric ligation decreases significantly (*p* < 0.01) the mucus production in the negative control compared with that in the normal control. The treatment with the extract (100, 200, and 400 mg/kg) significantly increased the mucus production in a dose-dependent manner (34.48; 38.64; and 65.30 mg) compared with the negative control (28.28 mg) (Table 2). Gastric acidity decreased significantly (*p* < 0.05) at 100 and 400 mg/kg (5.20 and 4.80 mEq/L, respectively) of extract compared with the negative control (8.40 mEq/L) (Table 3).

### 3.3. Effect of RBAEDM on Gastric Acid Secretion and Gastric Ulcers Induced by Association between Pyloric Ligation and Histamine

**Figure 2:**
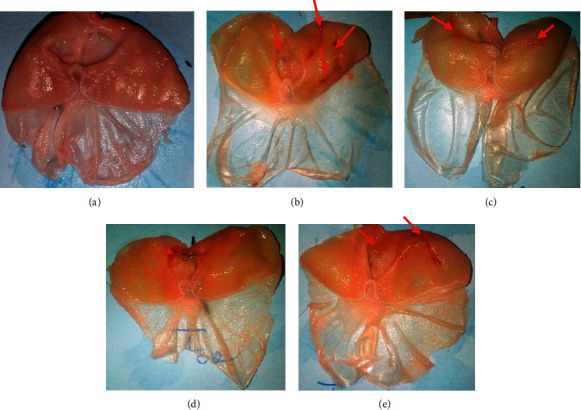
Photograph of stomach ulcerated by pyloric ligation combined with histamine. (a) Normal control; (b) negative control; (c) 200 mg/kg of RBAEDM; (d) 400 mg/kg of RBAEDM; and (e) 50 mg/kg of ranitidine; 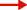: indications of gastric lesions.

The following photographs show the lesions of the gastric mucosa induced by the combination of pyloric ligation and histamine, and these appear as dark red bands (Figure 2). No lesion was observed in the normal control (Photograph A). The surface of these lesions is larger in the negative control (Photograph B: 28.40 mm^2^); this decreases significantly in animals treated at a dose of 200 and 400 mg/kg of extract and ranitidine (Photograph C: 22.20; Photograph D: 3.60; and Photograph E: 12.40 mm^2^, respectively). This decrease in ulcerated surface area is correlated with a significant (*p* < 0.01) increase in mucus production (78 mg) and a significant (*p* < 0.05) decrease in acidity (13.20 mEq/L) at the dose of 400 mg/kg compared with the negative control (48.02 mg and 24.40 mEq/L, respectively) (Tables 4 and 5). The percentage inhibition of pepsin activity increased by 38.79 and 43.10% at 200 and 400 mg/kg extract, respectively.

### 3.4. Effect of RBAEDM on Gastric Acid Secretion and Gastric Ulcers Induced by Pyloric Ligation Combined with Carbachol

**Figure 3:**
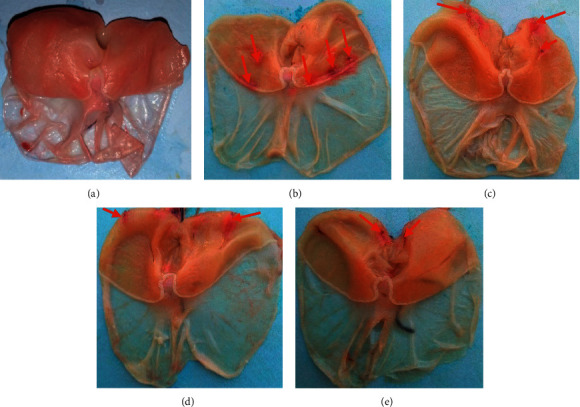
Photograph of stomach ulcerated by pyloric ligation combined with carbachol. (a) Normal control; (b) negative control; (c) 200 mg/kg of RBAEDM; (d) 400 mg/kg of RBAEDM; and (e) 50 mg/kg of verapamil; 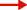: indications of gastric lesions.

Figure 3 shows stomach ulcerated by pyloric ligation combined with carbachol. The stomach wall of normal control does not show any ulcer (Photograph A). It can be seen that the surface area of these lesions is larger in the negative control (Photograph B: 22.60 mm^2^) and decreases in animals treated with the extract at doses of 200 and 400 mg/kg and verapamil (Photograph C: 8.80; Photograph D: 7.40; and Photograph E: 12.20 mm^2^, respectively). The treatment with RBEADM (200 and 400 mg/kg) significantly increased (*p* < 0.001) the mucus production (77.30 and 84.48 mg) and significantly decreased (*p* < 0.01) gastric acidity compared with the negative control (35.84 mg and 30 mEq/L, respectively) (Tables 6 and 7). Percentage inhibition of pepsin activity increased by 32.52 and 34.94% at 200 and 400 mg/kg.

### 3.5. Effect of RBAEDM on Some Oxidative Stress Parameters and Nitrites

Table 8 shows the effect of the aqueous extract on some parameters of oxidative stress. It shows a significant increase (*p* < 0.05) in the MDA level in the negative control compared with that in the normal control. On the other hand, the treatment with RBAEDM resulted in a significant decrease (*p* < 0.05; *p* < 0.01) in MDA levels in the extract-treated groups compared with that in the negative control. This decrease in MDA concentration correlates with a significant (*p* < 0.05; *p* < 0.01) increase in catalase activity. There was a significant (*p*˂0.001) and dose-dependent increase in the level of nitrite in stomach tissues at different doses of the extract.

### 3.6*. In Vitro* Antioxidant Effect of RBAEDM

*In vitro* antioxidant results show that the root bark aqueous extract of *Diospyros mespiliformis* has the ability to trap 77.7% of ABTS and 68.57% of DPPH and to reduce iron by 58.33% (Table 9). The concentrations of RBEADM inhibiting 50% (IC_50_) of the ABTS, DPPH radical, and FRAP were 220, 494, and 543 *μ*g/mL, respectively, compared with the butylhydroxytoluene (BHT) control of 175 *μ*g/mL (Table 9).

## 4. Discussion

Gastric ulcer can be caused by gastric acid hypersecretion [33]. Acid hypersecretion can result either from stimulation of histamine, gastrin, and acetylcholine receptors, or from uncontrolled production of gastrin in the case of Zollinger–Ellison syndrome [34]. In this work, hypersecretion was induced by pyloric ligation and by the combination of pyloric ligation with histamine and then carbachol.

Pyloric ligation induces basal acid secretion and was performed for antisecretory screening purpose. Accumulation of acid in the stomach is the cause of lesions. In fact, hydrochloric acid (HCl) causes lesions either by direct irritation of the stomach membrane cells that cause stomach membrane necrosis or by activating the conversion of pepsinogen into pepsin, which is a proteolytic enzyme that destroys membrane proteins [4, 35]. In addition, pyloric ligation and laparotomy also induce stress, which contributes to acid secretion, thus aggravating the gastric mucosa damaging. RBAEDM significantly and dose-dependently decreased gastric acidity at all doses compared with the negative control. This decrease in acidity was correlated with a significant increase in pH at different doses of the extract compared with the negative control. These results would suggest that RBAEDM possesses an antisecretory activity. The work of [36] showed that the reduction in gastric acidity by the aqueous extract of *Corchorus olitorius* could involve either direct inhibition of acid secretion or a simple neutralization of the acid secreted by the parietal cells, hence the interest in exploring its antisecretory mechanism of action.

Elucidation of antisecretory mechanisms has been carried out using pyloric ligation associated with histamine and carbachol. Indeed, histamine is an agonist of the H_2_ receptors of parietal cells of the stomach. Histamine binding to the H_2_ receptors activates adenylate cyclase, leading to the synthesis of cAMP. The latter will phosphorylate the H^+^/K^+^/ATPase pump and consequently stimulate the secretion of gastric acid [37]. Carbachol is a cholinergic receptor agonist, which binds to the muscarinic type 3 receptors (M_3_). This binding activates phospholipase C, which catalyzes the synthesis of phosphatidylinositol triphosphate (IP_3_) and diacylglycerol (DAG), which causes an increase in the concentration of cytosolic calcium [33] and consequently leads to the phosphorylation of the H^+^/K^+^/ATPase pump and thus the secretion of gastric acid. Chemical agents that decrease gastric acid secretion via blockade of H_2_ and M_3_ receptors are important in the treatment of gastric ulcers involving gastric acid hypersecretion [38]. Thus, receptor antagonists such as ranitidine and verapamil block their receptors and therefore cause non-phosphorylation of the H^+^/K^+^/ATPase pump. The subcutaneous injection of histamine resulted in an increase in gastric acidity, similarly to those injected with carbachol. RBAEDM administration decreased gastric acidity in animals given histamine and those given carbachol. The reduction in acidity by RBAEDM is accompanied by an increase in pH. The percentage of pepsin hydrolysis increases in rats treated with histamine and those treated with carbachol. These results would suggest that RBAEDM would have acted similarly to ranitidine and verapamil by reducing gastric acid secretion through a mechanism that would involve both the cholinergic and histaminergic pathways. Similar results were obtained by [4] who suggested that the aqueous extract of *Eremomastax speciosa* contains compounds that would act separately by both pathways or synergically to inhibit gastric acid secretion. The decrease in gastric acidity is thought to be attributed to flavonoids, which decrease histamine secretion by mast cells following inhibition of histidine decarboxylase [39].

Psychological stress, in addition to physical stress such as surgery, leads to oxidative stress in the stomach. Free radicals are normally produced during a normal cellular metabolism. Indeed, the production of free radicals leads to the oxidative stress, which results in the release of MDA. MDA is a classic marker of lipid peroxidation in stomach tissue [1]. SOD, CAT, and GSH are enzymes involved in protecting the stomach from free radical damage [40]. SOD converts the superoxide anion into H_2_O_2_, which is degraded into H_2_O and O_2_ by catalase [41]. RBAEDM induced a decrease in MDA levels correlated with the increase in activity of antioxidant enzymes such as SOD, CAT, and GSH at different doses of the extract. In this study, a significant decrease in MDA rate was correlated with an increase in SOD and CAT activity. The decrease in lipid peroxidation by the extract would suggest the *in vivo* antioxidant capacity of RBAEDM. Our results are similar to those obtained by [6] who had shown that the aqueous extract of *Emilia praetermissa* decreased the MDA level in gastric tissue and therefore decreased lipid peroxidation.

The confirmation of the antioxidant activity *in vivo* was made by antiradical tests with ABTS, DPPH, and FRAP from RBAEDM. In addition, RBAEDM has the ability to trap the ABTS and DPPH radicals and to reduce iron FRAP. This further confirms the observed *in vivo* antioxidant capacity of the extract. The IC_50_ of the aqueous extract of the leaves of *Diospyros mespiliformis* studied in Benin was 0.58 mg/mL [15]. This value is higher than that of our extract and shows that the Benin sample is less active than the one studied in this work. This observed activity would be due to the extract's richness in polyphenols and flavonoids, which are powerful free radical scavengers [42].

The mucus-bicarbonate barrier is the first line of defense against gastric acid secretion and is under the control of endogenous prostaglandins, which are involved in the protection of the gastric epithelium [16]. The treatment with RBAEDM significantly increased the mucus production in a dose-dependent manner in all three models. The observed action would be due to the presence of flavonoids, which reinforce the defense of the gastric mucosa by direct stimulation of the gastric secretion of mucus; the tannins lead to the precipitation of proteins at the site of the ulcer forming an impermeable barrier against gastric acid. Saponins stimulate mucus-producing factors in the gastric mucosa [16, 43].

Nitric oxide (NO) plays a protective role in the gastric mucosa *via* several mechanisms. It helps in the regulation of gastric acid production and mucus secretion by activating soluble guanylate cyclase, increases mucosal blood flow, binds to ß_3_ adrenergic receptors [44], and brings oxygen and nutrients into the mucosa while removing harmful waste products. RBAEDM significantly and dose-dependently increased nitrite levels at all the doses. These results suggest that RBAEDM can act by stimulating endothelial cells to release NO, which is gastroprotective. Our results are in line with those obtained by [45] who showed that the methanolic extract of *Distemonanthus benthamianus* plays its cytoprotective role by increasing NO level in gastric tissues.

## 5. Conclusion

RBAEDM inhibits ulcer formation by stimulating mucus production, enhancing antioxidant status, and inhibiting acid secretion. Antisecretory property could result from its action at the level of cholinergic and histaminergic pathways, and this thanks the presence of pharmacologically active phytoconstituents. The results obtained from this study justify its use in ethnopharmacology for the treatment of gastric ulcers.

## Figures and Tables

**Table 1:** Quantitative phytochemical analysis of RBAEDM.

Polyphenols (mEqAG/100g)	Flavonoids (mEqQu/100 g)	Tannins (mEqCa/100 g)	Anthocyanins (mEqCy-3-G/100 g)	Saponins (mEqG/100 g)
86.58 ± 0.73	55.22 ± 0.83	21.71 ± 0.20	10.14 ± 0.57	21.92 ± 0.35

mEqAG: milliequivalent gram of gallic acid, mgEqQu: milliequivalent gram of quercetin, mEqCa: milliequivalent gram of catechin, mEqCy-3-G: milliequivalent gram of cyanidin-3-glucoside, and mEqG: milliequivalent gram of galactose.

**Table 2:** Effect of RBAEDM on ulcers induced by pyloric ligation.

Treatments	Dose (mg/kg)	Ulcerated surface (mm^2^)	Ulcer index	Inhibition (%)	Mucus mass (mg)
Normal control	−	−	−	−	42.67 ± 2.25
Negative control	−	22.40 ± 5.53	4.24 ± 0.51	−	28.28 ± 1.52^##^
RBAEDM	100	5.60 ± 0.81^∗∗^	2.56 ± 0.19	39.62	34.48 ± 2.23
RBAEDM	200	8.80 ± 2.63^*∗*^	3.00 ± 0.75	29.24	38.64 ± 2.83^*∗*^
RBAEDM	400	7.60 ± 2.94^*∗*^	2.35 ± 0.58	44.57	65.30 ± 3.83^∗∗∗^
Ranitidine	50	8.00 ± 1.30^*∗*^	2.78 ± 0.34	34.43	34.12 ± 1.62

*N* = 5: number of animals per group. Values are expressed as mean ± standard error of the mean; RBAEDM: root bark aqueous extract of *Diospyros mespiliformis*;  ^*∗*^*p* < 0.05,  ^*∗*^ ^*∗*^*p* < 0.01, and  ^*∗*^ ^*∗*^ ^*∗*^*p* < 0.001: significantly different compared with the negative control; ^##^*p* < 0.01: significantly different compared with the normal control.

**Table 3:** Effect of RBAEDM on gastric acid secretion induced by pyloric ligation.

Treatments	Dose (mg/kg)	VGJ (mL)	pH	Gastric acidity (mEq/L)
Normal control	−	−	−	−
Negative control	−	1.92 ± 0.19	2.46 ± 0.15	7.76 ± 0.14
RBAEDM	100	1.28 ± 0.21^*∗*^	3.69 ± 0.37^*∗*^	4.48 ± 0.42^*∗*^
RBAEDM	200	0.84 ± 0.09^∗∗∗^	4.38 ± 0.09^∗∗^	4.32 ± 0.42^*∗*^
RBAEDM	400	0.66 ± 0.08^∗∗∗^	4.25 ± 0.32^∗∗^	4.00 ± 0.50^*∗*^
Ranitidine	50	0.67 ± 0.13^∗∗∗^	6.08 ± 0.54^∗∗∗^	6.16 ± 1.55

*N* = 5: number of animals per group. Values are expressed as mean ± standard error of the mean; RBAEDM: root bark aqueous extract of *Diospyros mespiliformis*; VGJ: volume of gastric juice;  ^*∗*^*p* < 0.05,  ^*∗*^ ^*∗*^*p* < 0.01, and  ^*∗*^ ^*∗*^ ^*∗*^*p* < 0.001: significantly different compared with the negative control.

**Table 4:** Effect of RBAEDM on PL-induced ulcers combined with histamine.

Treatments	Dose (mg/kg)	Ulcerated surface (mm^2^)	Ulcer index	Inhibition (%)	Mucus mass (mg)
Normal control	−	−	−	−	42.67 ± 2.25
Negative control	−	28.40 ± 2.46	4.63 ± 0.18	−	48.02 ± 3.60
RBAEDM	200	20.20 ± 1.39^∗∗^	4.19 ± 0.13^∗∗^	9.50	49.14 ± 3.79
RBAEDM	400	3.60 ± 1.50^∗∗∗^	2.20 ± 0.20^∗∗∗^	52.48	72.63 ± 2.65^∗∗^
Ranitidine	50	12.40 ± 1.36^∗∗∗^	3.23 ± 0.34^∗∗^	30.23	46.86 ± 6.82

*N* = 5: number of animals per group. Values are expressed as mean ± standard error of the mean; RBAEDM: root bark aqueous extract of *Diospyros mespiliformis*;  ^*∗*^ ^*∗*^*p* < 0.01 and  ^*∗*^ ^*∗*^ ^*∗*^*p* < 0.001: significantly different compared with the negative control.

**Table 5:** Gastric acid hypersecretion induced by pyloric ligation combined with histamine.

Treatments	Dose (mg/kg)	VGJ (mL)	pH	Gastric acidity (mEq/L)	IPA (%)
Normal control	−	−	−	−	−
Negative control	−	2.68 ± 0.53	2.70 ± 0.37	24.40 ± 2.92	−
RBAEDM	200	0.90 ± 0.40^*∗*^	4.40 ± 0.88	15.60 ± 2.40	38.79
RBAEDM	400	0.64 ± 0.18^∗∗^	5.81 ± 0.51^∗∗^	13.20 ± 3.00^*∗*^	43.10
Ranitidine	50	1.58 ± 0.22^*∗*^	2.55 ± 0.18	19.60 ± 1.60	22.41

*N* = 5: number of animals per group. Values are expressed as mean ± standard error of the mean; RBAEDM: root bark aqueous extract of *Diospyros mespiliformis*; VGJ: volume of gastric juice, IPA: inhibition of pepsin activity; ^*∗*^*p* < 0.05 and ^∗∗^*p* < 0.01: significantly different compared with the negative control.

**Table 6:** Effects of RBAEDM on gastric ulcers induced pyloric ligation associated with carbachol.

Treatments	Dose (mg/kg)	Ulcerated surface (mm^2^)	Ulcer index	Inhibition (%)	Mucus mass (mg)
Normal control	−	−	−	−	42.67 ± 2.25
Negative control	−	22.60 ± 1.28	4.67 ± 0.28	−	35.84 ± 3.55
RBAEDM	200	8.80 ± 0.80^∗∗∗^	2.58 ± 0.30^∗∗∗^	44.75	77.30 ± 4.11^∗∗∗^
RBAEDM	400	7.40 ± 0.60^∗∗∗^	1.89 ± 0.10^∗∗∗^	59.52	84.48 ± 2.55^∗∗∗^
Verapamil	50	12.20 ± 0.66^∗∗∗^	2.29 ± 0.15^∗∗∗^	50.96	39.52 ± 1.25

*N* = 5: number of animals per group. Values are expressed as mean ± standard error of the mean; RBAEDM: root bark aqueous extract of *Diospyros mespiliformis*; ^∗∗∗^*p* < 0.001: significantly different compared with the negative control.

**Table 7:** Gastric acid hypersecretion induced by pyloric ligation associated with carbachol.

Treatments	Dose (mg/kg)	VGJ (mL)	pH	Gastric acidity (mEq/L)	IPA (%)
Normal control	−	−	−	−	−
Negative control	−	3.16 ± 0.25	1.92 ± 0.13	30 ± 3.34	−
RBAEDM	200	2.60 ± 0.54	2.09 ± 0.22	18.40 ± 0.74^∗∗^	32.52
RBAEDM	400	1.88 ± 0.54	2.47 ± 0.36	18.00 ± 0.63^∗∗^	34.95
Verapamil	50	1.80 ± 0.53	3.42 ± 0.85	21.20 ± 2.33^∗∗^	12.19

*N* = 5: number of animals per group. Values are expressed as mean ± standard error of the mean; RBAEDM: root bark aqueous extract of *Diospyros mespiliformis*; VGJ: volume of gastric juice; IPA: inhibition of pepsin activity; ^∗∗^*p* < 0.01: significantly different compared with the negative control.

**Table 8:** Effect of RBAEDM on some parameters of oxidative stress and nitrites.

Treatment	Dose (mg/kg)	MDA (*μ*mol/mg protein)	SOD (U/mg protein)	CAT (*μ*mol H_2_O_2_/min/mg protein)	GSH (mmol/g protein)	Nitrites (mol/L)
Normal control	−	2.03 ± 0.52	225.55 ± 4.15	80.09 ± 5.36	4.23 ± 5.14	5.01 ± 0.86
Negative control	−	2.50 ± 0.10^#^	214.00 ± 6.83	77.10 ± 4.12	4.59 ± 4.39	5.56 ± 0.37
RBAEDM	100	2.11 ± 0.68^*∗*^	224.00 ± 3.23	79.23 ± 1.28	5.83 ± 1.58	8.10 ± 0.98^∗∗^
RBAEDM	200	2.14 ± 0.10^*∗*^	229.60 ± 2.00	91.27 ± 1.93^*∗*^	5.90 ± 2.05	8.36 ± 0.62^∗∗^
RBAEDM	400	1.97 ± 0.92^∗∗^	221.80 ± 4.18	96.30 ± 5.81^∗∗^	5.00 ± 1.73	9.94 ± 0.92^∗∗∗^
Ranitidine	50	2.06 ± 0.12^*∗*^	216.60 ± 4.70	87.87 ± 4.35^*∗*^	5.79 ± 4.27	5.17 ± 0.16

*N* = 5: number of animals per group. Values are expressed as mean ± standard error of the mean; RBAEDM: root bark aqueous extract of *Diospyros mespiliformis*; MDA : malondialdehyde; SOD : superoxide dismutase; CAT : catalase; GSH : reduced glutathione; ^*∗*^*p* < 0.05, ^∗∗^*p* < 0.01, and ^∗∗∗^*p* < 0.001: significantly different compared with the negative control; ^#^*p* < 0.05: significantly different compared with normal control.

**Table 9:** *In vitro* antioxidant capacity of RBAEDM.

Antioxidants	ABTS	DPPH	FRAP
%I	77.70 ± 0.72	68.57 ± 0.66	58.33 ± 0.54
IC_50_ (*μ*g/mL)	220	494	543

%*I*: percentage of inhibition; IC_50_: inhibitory concentration of 50%.

## Data Availability

The data used to support the findings of this study are available from the corresponding author upon request.
